# Psychometric Properties of the Arabic Version of the Problem Areas in Diabetes Scale in Primary Care

**DOI:** 10.3389/fpubh.2022.843164

**Published:** 2022-02-25

**Authors:** Hazem A. Sayed Ahmed, Samar Farag Mohamed, Sally Fawzy Elotla, Mona Mostafa, Jaffer Shah, Ahmed Mahmoud Fouad

**Affiliations:** ^1^Department of Family Medicine, Faculty of Medicine, Suez Canal University, Ismailia, Egypt; ^2^Department of Public Health, Occupational and Environmental Medicine, Faculty of Medicine, Suez Canal University, Ismailia, Egypt; ^3^Department of Internal Medicine, Faculty of Medicine, Suez Canal University, Ismailia, Egypt; ^4^Medical Research Center, Kateb University, Kabul, Afghanistan

**Keywords:** diabetes-distress, PAID, primary healthcare, type 2 diabetes, Arabic

## Abstract

**Background:**

The Problem Areas in Diabetes (PAID) scale is a reliable and valid tool that is widely used for diabetes-distress screening, but the Arabic version of the scale lacks validity and reliability analysis in primary healthcare (PHC) patients. Our study aimed to evaluate the psychometric properties of the Arabic version of the PAID (AR-PAID) scale among Egyptian patients with type 2 diabetes mellitus (T2DM) in PHC settings.

**Methods:**

We conducted a cross-sectional study on a convenience sample of 200 patients from six rural PHC settings in the Ismailia governorate. The confirmatory factor analysis (CFA) was performed to test the goodness-of-fit to the predefined models of the PAID. Convergent construct was evaluated through correlations with the Arabic versions of the Patient Health Questionnaire 9 (PHQ-9), Generalized Anxiety Disorder Scale (GAD-7), and 5-item World Health Organization Well-Being Index (WHO-5), additionally glycated hemoglobin (HbA1c) levels. Discriminant validity was evaluated through associations with patients' sociodemographic and clinical characteristics. Reliability was evaluated through internal consistency (Cronbach's α) and test-retest reliability analysis (intraclass correlation coefficient, ICC).

**Results:**

The CFA demonstrated the best fit for a four-factor model. The AR-PAID was significantly correlated with the following measures: PHQ-9 (rho = 0.71, *p* < 0.001), GAD-7 (rho = 0.50, *p* < 0.001), WHO-5 (rho = −0.69, *p* < 0.001), and HbA1c (rho = 0.36, *p* < 0.001), supporting sound convergent validity. Discriminant validity was satisfactory demonstrated. Internal consistency was excellent (α = 0.96) and test-retest reliability was stable (ICC = 0.97).

**Conclusions:**

The AR-PAID scale is a valid and reliable instrument for diabetes-distress screening in primary care patients with T2DM that can be used in clinical settings and research. Further research is needed to validate short forms of the AR-PAID scale.

## Introduction

Diabetes is a global health problem with a prevalence of 9.3% in 2019. Its prevalence in the Middle East and North Africa region was the highest age-adjusted diabetes prevalence (12.2%) of all world regions. T2DM accounts for about 90% of all diabetes cases worldwide (1).

Diabetes-distress is a common comorbid psychological problem with diabetes (36%) (2), and it reflects the diabetic patient's emotional response to the burden of living with debilitating complications and the greater self-care demands of diabetes (3–6). Diabetes-distress is linked to suboptimal adherence and poor glycemic outcomes (5, 7–10). The American Diabetes Association has recommended screening for diabetes-distress by validated tools at the initial visit of a diabetic patient, at periodic intervals, and when there is a change in disease, treatment, or life circumstances (11).

The PAID scale is an instrument widely used to assess diabetes-distress. It includes 20 items (2). Polonsky et al. had developed this scale for assessing psychosocial adjustment to diabetes in the USA (12–14). Hermanns et al. found that the PAID scale may also be used as a sound-screening instrument for depression in diabetes with a cut-off score of ≥33, which is lower than the normal cut-off score of ≥40 for identifying diabetes-distress (15). The PAID scale is also a useful instrument for assessing several aspects of quality of life in diabetes patients (16, 17). The responsiveness of the PAID has also been found; the PAID is able to detect change as demonstrated in seven diabetes intervention studies (18).

Interest in diabetes-distress across countries through the previous two decades means that versions in multiple languages of the PAID instruments are not only desirable, but also necessary in order to compare the findings about diabetes-distress from different languages, cultures, and societies, in addition to the improvement of diabetic care. The original PAID has been translated into several languages successfully, and these versions of it are reliable and valid instruments (19–32).

Previous Arabic studies evaluated the validity and reliability of the 20-item AR-PAID scale in Kuwait and Lebanon. An early study revealed that the AR-PAID was valid and reliable in older adult patients with T2DM, who were recruited from one hospital-based diabetes unit and one primary diabetic clinic in Kuwait City. Another Arabic study found that the AR-PAID had excellent reliability and an acceptable convergent with HbA1c in Lebanese adults with T2DM, but its construct validity was not assessed (33, 34).

Venkataraman et al. displayed the abridged 16-item PAID scale as reliable, valid, and sensitive in Singaporean patients with diabetes (35). Hsu et al. showed that the 8-item short form of the PAID demonstrated sound validity and reliability in Chinese patients with T2DM (36). McGuire et al. developed the 5-item PAID (PAID-5) and 1-item PAID (PAID-1) from the original PAID (37).

In light of the burden of T2DM in Middle East and Arabic countries, increased interest in diabetes-distress, and a lack of studies in validity and reliability of the AR-PAID in primary care, this study was carried out to evaluate the psychometric properties of the AR-PAID in Egyptian patients with T2DM attending PHC settings.

## Methods

### Design, Sampling, and Setting

A cross-sectional study was conducted on a convenience sample of patients with T2DM from September 2020 to June 2021. The participants were recruited from six rural PHC settings at the Ismailia governorate, affiliated with Egypt's Ministry of Health and Population. Soper's online calculator of a sample size for structural equation models was used to estimate the sample size for a CFA model of a four-factor and the 20 observed variables of the PAID scale (38). The sample size of 173 was enough to detect an expected effect size of 0.3 (a medium effect size) at 5% alpha error and 90% power of the study. And in addition, more increase of the sample size to 200 patients to compensate for dropout, guided by the “10 participants per item” rule of thumb for calculation of the sample size for confirmatory factor analysis (39).

We included participants aged 18 years or older who were diagnosed with T2DM for at least 1 year. An informed consent was obtained from every participant. We excluded patients with a severe mental illness, cognitive impairment, or gestational diabetes. Face-to-face interviews by the second author were used to collect data. A 1-month test-retest on reliability of the AR-PAID scale was conducted on 50 participants.

### Study Measures and Scales

Our study's questionnaire consisted of several parts. The initial part included the sociodemographic and clinical characteristics, e.g., age, gender, marital status, family income, occupation, smoking diabetes duration, diabetes treatment, co-morbidities, and diabetes-related complications (e.g., cardiovascular, cerebrovascular, retinopathy, nephropathy, neuropathy, or peripheral vascular complications). The next part of our study's questionnaire included the Arabic versions of the PAID, PHQ-9 (40, 41), GAD-7 (41, 42), and WHO-5 (43, 44) scales.

The original PAID scale was written in English and consisted of 20 items rated on a 5-point Likert scale from 0 to 4, with 0 indicating no problem and 4 indicating a major problem. The PAID calculates a total score range of 0 to 100 by adding the replies to all 20 items and multiplying the result by 1.25. The higher the score, the more diabetes-distress, with a score of 40 or above indicating significant diabetes-distress (15, 45). The AR-PAID was obtained from the Joslin Diabetes Center.

The PHQ-9 consists of 9 items, each with a score ranging from 0 (never) to 3 (almost every day), for a total score of 0 to 27. For the detection of major depressive disorder, a cut-off value of ≥10 has an 88 percent sensitivity and an 88 percent specificity (40). The Arabic version of the PHQ-9 has previously shown to be a valid and reliable tool to screen depression in a Saudi sample. Cronbach's alpha was 0.857. Inter-item correlations range between 0.177 and 0.648 (41).

The GAD-7 is a 7-item anxiety portion that is part of the full GAD. Each GAD item is given a score ranging from 0 (never) to 3 (almost every day), for a total score of 0 to 21. GAD is indicated by a cut-off value ≥10 (89 percent sensitivity and 82 percent specificity) (42). The Arabic version of the GAD-7 demonstrated a sound validity and reliability properties. Cronbach's alpha was 0.763. Its range of the inter-item correlations was from 0.204 to 0.426. (41).

The WHO-5, which was generated from the WHO-10 (43), is one of the most extensively used questionnaires for assessing subjective psychological wellbeing (46). This scale only has elements that are positively stated. On a 6-point Likert-type scale ranging from 0 (not present) to 5 (constantly present), the degree to which these feelings were present in the previous 2 weeks is scored. The raw score is multiplied by 4 to convert the item scores to a 0–100 scale (47). The Arabic version of the WHO-5 demonstrated validity and reliability in the Lebanese older population (44).

All participants had their anthropometric measurements taken, including their body weight (in kg) and height (in cm). The BMI was computed by dividing the body weight (in kg) by the squared root of the height (in meters). Overweight participants had a BMI of 25–29.9, while obese participants had a BMI of more than 30 kg/m^2^. The patient's most recent HbA1c readings were recorded (<8 weeks prior or 12 weeks after the interview). Adult and older adult patients with controlled glycemic targets were identified by HbA1c levels <7 and 7.5%, respectively (11).

### Statistical Analysis

Data management and analyses were performed using the Statistical Package for the Social Sciences (SPSS), version 25.0 (IBM Corporation, NY, USA). Categorical variables were tabulated as frequencies and percentages, while continuous variables were summarized by means and standard deviations (median and interquartile ranges, if not normally distributed). Continuous variables were tested for data normality with the Kolmogorov-Smirnov test.

The AR-PAID was tested for both reliability and validity (construct, convergent, and discriminant). Reliability testing was performed using Cronbach's alpha for internal consistency, as well as ICC for test-retest reliability.

The construct validity of AR-PAID-20 was evaluated in a series of confirmatory factor analyses (CFA), in which we compared the model fit indices of previously proposed models of PAID's factor structure models: the original one-factor of the original PAID, two-factor of the Turkish PAID, three-factor of the Swedish PAID, four-factor of the Dutch PAID, and four-factor of the Spanish PAID (13, 19, 22, 24, 28). The model fit included: (1) the ratio of chi-square statistics [χ^2^] to the degrees of freedom [df] (CMIN/DF) and associated *p*-values; (2) the standardized root mean square residual (SRMR); (3) the root mean squared error of approximation (RMSEA); (4) the comparative fit index (CFI); and (5) the Tucker Lewis Index (TLI). The model fit acceptability was set at CMIN/DF <3, SRMR and RMSEA ≤ 0.08, and CFI and TLI ≥0.90 (27). All CFAs were performed with Mplus software for statistical analysis with Latent variables, version 7.4 (Los Angeles, CA, USA: Muthén and Muthén (48)].

The convergent validity was established by testing for correlations between the AR-PAID and other related scales (e.g., the PHQ-9 for depression and the GAD-7 for anxiety) and the level of glycemic control (HbA1C level). Spearman's correlation was used with the following coefficient values: 0–0.19 considered very weak, 0.2–0.39 considered weak, 0.40–0.59 considered moderate, 0.6–0.79 considered strong, and 0.8–1.0 considered very strong correlations (49).

In regards to discriminant validity, the AR-PAID's ability to differentiate between different groups of patients was tested with the Mann–Whitney and Kruskal–Wallis tests, in which the number of groups compared was given. Total AR-PAID scores were compared across patients' groups, such as patients with major depression or generalized anxiety disorder, patients with poor glycemic control, and patients with different demographic and clinical characteristics. All *p* < 0.05 were considered statistically significant at a 95% level of confidence.

## Results

### Descriptive Statistics

Two-hundred patients with T2DM were interviewed in this study. Participants' ages ranged from 20 to 80 years, with a mean of 48.3 ± 11.4 years. Twenty-four percentage of them were younger than 40 years. Females made up 60% of all participants. The majority (76%) of the participants were married. The mean duration of diabetes was 8.2 ± 6.2 years and ranged from 1 to 30 years. Sixty-seven (33.5%) patients were on insulin-containing regimen. Most (64.5%) of the participants had one or more diabetes-related complications, which were neuropathy (50.5%), retinopathy (36.5%), foot problems (33.0%), and nephropathy (21.5%). The most common chronic comorbidities were obesity (36.5%) and hypertension (23.5%). The mean HbA1c level was 7.9 ± 0.84% and ranged from 6 to 14.5%, with only 17 patients (8.5%) having achieved good glycemic control (Table 1).

**Table 1:** Patients' demographic and clinical characteristics (*N* = 200).

**Characteristics**	**Frequency (%)**
**Age (years), mean** **±SD (range)**	48.8 ±12.1 (20–80)
<40 years	48 (24.0%)
40–59	107 (53.5%)
60+	45 (22.5%)
**Gender**	
Female	120 (60.0%)
Male	80 (40.0%)
**Marital status**	
Single	8 (4.0%)
Married	152 (76.0%)
Divorced/widow	40 (20.0%)
**Education level**	
Illiterate	44 (22.0%)
Less than secondary	7 (3.5%)
Secondary	117 (58.5%)
University and above	32 (16.0%)
**Work status**	
Not employed/housewives	92 (46.0%)
Employed/business owners/freelancers	83 (41.5%)
Retired	25 (12.5%)
**Duration of diabetes, mean** **±SD (range)**	8.2 ± 6.2 (1–30)
<5 years	67 (33.5%)
5–10 years	82 (41.0%)
More than 10 years	51 (25.5%)
**Type of antidiabetic medications**	
Oral hypoglycemics	133 (66.5%)
Insulin-Containing regimens	67 (33.5%)
**Number of diabetes-related complications**	
None	71 (35.5%)
Single	49 (24.5%)
Two or more	80 (40.0%)
**Type of diabetes-related complications**	
Retinopathy	73 (36.5%)
Nephropathy	43 (21.5%)
Neuropathy	101 (50.5%)
Foot problems	66 (33.0%)
Others	12 (6.0%)
**Other chronic comorbidities**	
Obesity	73 (36.5%)
Hypertension	47 (23.5%)
Dyslipidemia	22 (11.0%)
**HbA1c %, mean** **±SD (range)**	7.9 ± 0.84 (6.0–14.5)
**Glycemic control**	
Controlled	17 (8.5%)
Uncontrolled	183 (91.5%)

### Factor Structure of the AR-PAID

Five alternative factor structures were compared for the goodness-of-fit indices using confirmatory factor analysis in Table 2. The Belendez's et al. revised four-factor (28) showed the best goodness-of-fit indices (CMIN/DF = 2.26, RMSEA = 0.079, SRMR = 0.057, CFI = 0.099, and TLI 0.988). Accordingly, the factor structure of the Belendez's et al. model (28) is further explained with standardized factor loadings in Table 3. The standardized factor loadings for items in this model were satisfactory and statistically significant and ranged from 0.526 to 1.002.

**Table 2:** Comparisons of fit indices of different AR-PAID factor solutions (*N* = 200).

**Factor solution models**	**Factors (items included)**	**Goodness-of-Fit indices**						
		**DF**	**X^**2**^**	**CMIN/DF**	**RMSEA**	**CFI**	**TLI**	**SRMR**
One-Factor (13)	Problem areas in diabetes (items 1–20)	164	714.9^**^	4.36	0.127	0.972	0.969	0.078
Two-Factor (24)	• Diabetes distress (15 items): 1–14, 19 • Lack of support (5 items): 15–18, 20	164	678.4^**^	4.14	0.123	0.974	0.971	0.077
Three-Factor (22)	• Emotional problems (15 items): 3, 6–10, 12–14, 16, 19, 20 • Treatment problems (2 items): 1, 2 • Lack of support (3 items): 15, 17, 18	164	481.0^**^	2.93	0.097	0.984	0.982	0.059
Four-Factor (19)	• Emotional problems (12 items): 3, 6–10, 12–14, 16, 19-20 • Treatment problems (3 items): 1, 2, 15 • Food-related problems (3 items): 4, 5, 11 • Lack of support (2 items): 17, 18	164	391.1^**^	2.38	0.083	0.988	0.987	0.059
Revised four-factor (28)	• Emotional problems (12 items): 3, 6–10, 12-14, 16, 19–20 • Treatment problems (2 items): 1, 2 • Food problems (3 items): 4, 5, 11 • Lack of support (3 items): 15, 17, 18	164	371.2^**^	2.26	0.079	0.990	0.988	0.057

*AR-PAID, Arabic version of the Problem Areas in Diabetes; CMIN/DF, ratio of chi-square [χ^2^] value to the degrees of freedom [df] (good if CMIN/DF <3); CFI, comparative fit index (good fit ≥0.90); TLI, Tucker Lewis Index (good if ≥ 0.90); SRMR, standardized root mean square residual (good fit ≤ 0.08); RMSEA, root mean square error of approximation (acceptable fit ≤ 0.08)*.

** *Statistically significant p (<0.001)*.

**Table 3:** Factor loadings of the revised 4-factor model of the AR-PAID (*N* = 200).

	**Factor structure of the AR-PAID**	**Factor loadings**		
**Factors**	**Items number and shortened content**	**Std. estimates**	**SE**	***p*-value**
Emotional problems	Q3: Feeling scared	0.937	0.015	0.000
	Q6: Feeling depressed	0.978	0.008	0.000
	Q7: Indistinguishable mood	0.884	0.024	0.000
	Q8: Overwhelmed by diabetes regimen	0.909	0.020	0.000
	Q9: Worry about low blood sugar reactions	0.789	0.028	0.000
	Q10: Feeling angry	0.950	0.012	0.000
	Q12: Worry about the future complications	0.852	0.023	0.000
	Q13: Feeling guilty if get off track with management	0.932	0.017	0.000
	Q14: Not accepting diabetes	0.795	0.057	0.000
	Q16: Taking too much mental and physical energy	0.920	0.014	0.000
	Q19: Coping with complications of diabetes	0.809	0.029	0.000
	Q20: Burned out by effort to manage diabetes	0.893	0.019	0.000
Treatment problems	Q1: No clear treatment goals	0.947	0.012	0.000
	Q2: Feeling discouraged with treatment plan	0.983	0.006	0.000
Food problems	Q4: Uncomfortable social situations involving eating	0.807	0.063	0.000
	Q5: Feelings of deprivation of food	0.927	0.014	0.000
	Q11: Concerned about food and eating	0.935	0.013	0.000
Lack of support	Q15: Feeling unsatisfied with diabetes physician	0.526	0.130	0.041
	Q17: Feeling alone with diabetes	0.882	0.049	0.000
	Q18: Feeling that friend/ family are not supportive	1.002	0.061	0.000
		**Goodness-of-Fit indices**
	Model fit, χ^2^ (df), *p*-value	371.2 (164), <0.001^*^	
	CMIN/DF (χ^2^/df)	2.26	
	CFI	0.990	
	TLI	0.988	
	SRMR	0.057	
	RMSEA	0.079	

*AR-PAID, Arabic version of the Problem Areas in Diabetes; CMIN/DF, ratio of chi-square [χ^2^] value to the degrees of freedom [df] (good if CMIN/DF <3); CFI, comparative fit index (good fit ≥ 0.90); TLI, Tucker Lewis Index (good if ≥0.90); SRMR, standardized root mean square residual (good fit ≤ 0.08); RMSEA, root mean square error of approximation (acceptable fit ≤ 0.08); SE, standard error*.

* *Statistically significant at p < 0.001*.

### Convergent Validity of the AR-PAID

Table 4 shows the median and interquartile range (IQR) of the AR-PAID and its subscales. Convergent validity was confirmed by significant moderate-to-strong correlations between the total scores of the AR-PAID with: the PHQ-9 scale for depression (rho = 0.71, *p* < 0.001), the GAD-7 scale for anxiety (rho = 0.50, *p* < 0.001), and the WHO-5 wellbeing index (rho = −0.69, *p* < 0.001). The total score of the AR-PAID also showed a significant but weak correlation with HbA1c levels (rho = 0.38, *p* < 0.001). The AR-PAID subscales showed significant correlations with all measures, with correlation coefficients ranging from 0.15 to 0.68. All AR-PAID subscales had their highest correlations with the PHQ-9 scale and WHO-5 wellbeing index, while their weakest correlations existed with HbA1C levels (Table 4).

**Table 4:** Correlations of the AR-PAID and its subscales with other measures of effect and HbA1c (*N* = 200).

**AR-PAID**	**Median (IQR)**	**Correlation coefficient** ^a^			
		**PHQ-9**	**GAD-7**	**WHO-5**	**HbA1c**
Total AR-PAID (0–100)	16.3 (6.3–33.8)	0.71^**^	0.50^**^	−0.69^**^	0.36^**^
Emotional problems (0–60)	11.3 (3.8–21.3)	0.68^**^	0.51^**^	−0.68^**^	0.37^**^
Treatment problems (0–10)	1.3 (0–5.0)	0.59^**^	0.37^**^	−0.54^**^	0.28^**^
Food problems (0–15)	2.5 (0–6.3)	0.60^**^	0.37^**^	−0.52^**^	0.26^**^
Lack of support (0–15)	0 (0–1.3)	0.41^**^	0.36^**^	−0.29^**^	0.15^*^

*AR-PAID, Arabic version of the Problem Areas in Diabetes; GAD-7, Generalized Anxiety Disorder Scale 7; HbA1c, Glycated hemoglobin; PHQ-9, Patient Health Questionnaire 9; WHO-5, 5-item World Health Organization Well-Being Index*.

a *Spearman's Correlation. IQR: Interquartile range*.

** *p < 0.001*,

* *p < 0.05*.

### Discriminant Validity of the AR-PAID

**Figure 1:**
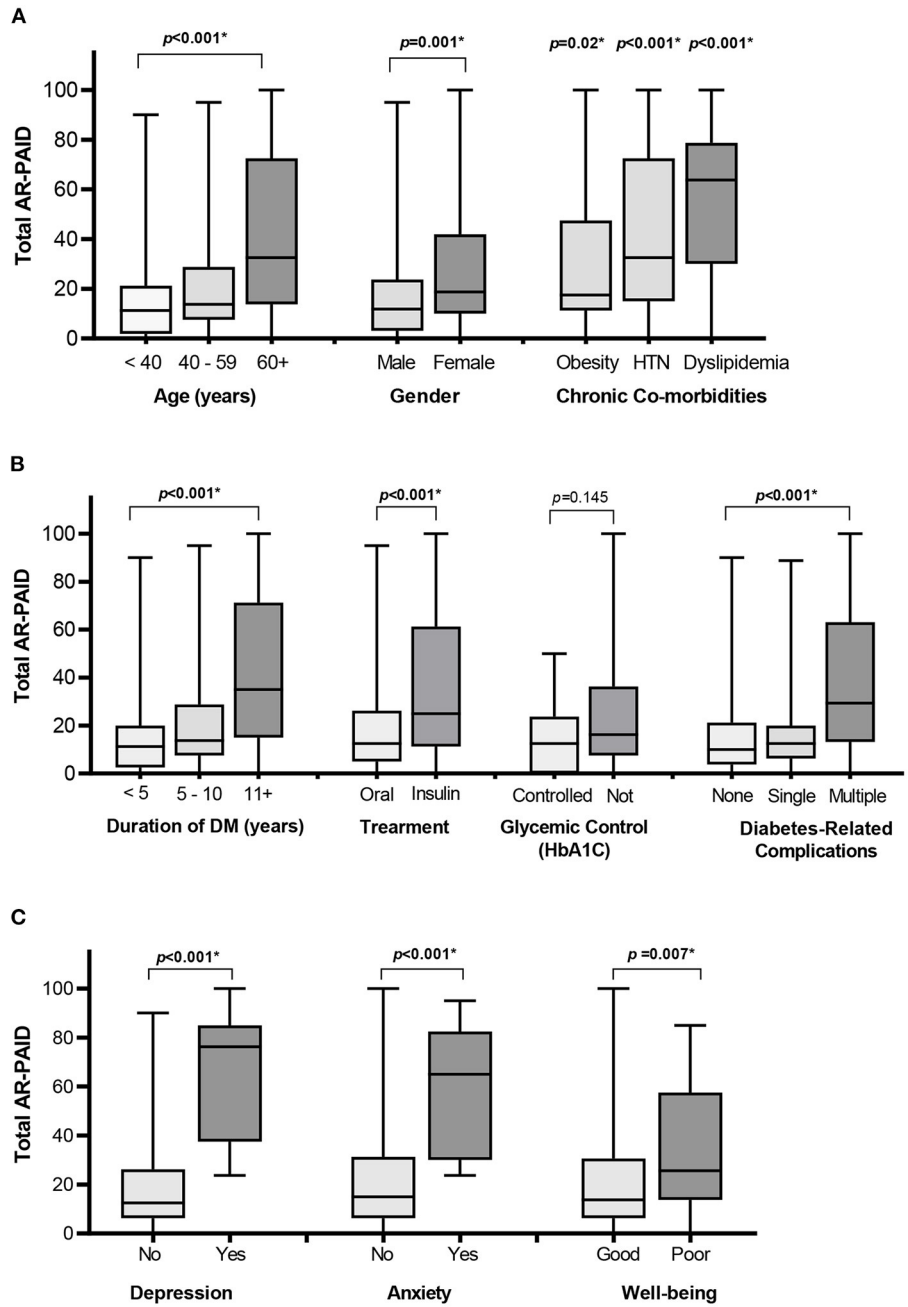
Distribution of total AR-PAID score by patients' demographic and clinical characteristics: age, gender, and co-morbidities **(A)**, diabetes-related characteristics **(B)**, and depression, anxiety, and WHO-wellbeing scales **(C)**. *Indicates a statistically significant *p* < 0.05. DM, diabetes mellitus; HTN, hypertension; WHO-5, 5-item World Health Organization Well-Being Index.

The AR-PAID scale showed the ability to discriminate between diabetes-distress among patients with different demographic and clinical characteristics (Figure 1). Female patients and patients with older age or longer disease duration showed significantly higher total scores of the AR-PAID. Patients on insulin-containing regimen also had significantly higher total AR-PAID scores compared to patients on oral hypoglycemic agents. Increasing the number of diabetes-related complications was significantly associated with higher total AR-PAID scores in contrast to those with no complications. Patients with obesity, hypertension, or dyslipidemia also showed significantly higher total AR-PAID scores compared to patients without comorbidities. In contrast, there was no significant difference in AR-PAID total scores between patients with controlled diabetes and those with uncontrolled (*p* = 0.145). Known-group validity was confirmed by the statistically significant differences in AR-PAID total scores between patients with and without depression (PHQ-9 ≥ 10), patients with and without generalized anxiety disorder (GAD-7 ≥ 10), and patients with and without poor wellbeing (WHO-5 index ≤ 50).

### Reliability of the AR-PAID

Cronbach's alpha for the total AR-PAID scale was 0.96, while it ranged from 0.65 to 0.96 for the AR-PAID subscales. The subscales of emotional and treatment problems showed the highest Cronbach's alpha. Test-retest reliability was investigated in 50 patients and revealed satisfactory significant ICCs. The total AR-PAID scale had an ICC of 0.97 (95% CI: 0.61–0.83, *p* < 0.001), while the ICC of the AR-PAID subscales ranged from 0.92 to 0.97 (Table 5).

**Table 5:** Internal consistency and test-retest reliabilities of the AR-PAID (*N* = 200).

**Items of the AR-PAID (score range)**	**Number of items**	**Cronbach α**	**Test-Retest reliability (*****n*** **=** **50)**	
			**ICC**	**95% CI**
Emotional problems (0–60)	12	0.96	0.93	0.86–0.96
Treatment problems (0–10)	2	0.94	0.97	0.88–0.99
Food problems (0–15)	3	0.79	0.92	0.52–0.97
Lack of support (0–15)	3	0.65	0.96	0.89–0.98
Total AR-PAID (0–100)	20	**0.96**	**0.97**	**0.94–0.98**

*AR-PAID, Arabic version of the problem areas in diabetes; SD, Standard deviation; ICC, intra-class correlation; CI, confidence interval*.

## Discussion

We evaluated the psychometric properties of the AR-PAID in Egyptian primary care patients with T2DM. We found that the AR-PAID had a four-factor structure, had a satisfied convergent and discrimination validity, and was internally consistent with stable test-retest reliability.

A CFA supported a four-factor structure of the AR-PAID similar to those found in the Spanish and Kinyarwanda versions of the PAID (28, 31). A CFA of the AR-PAID also demonstrated marginal fit to the four-factor model of the Duch PAID, as well as the three-factor model of the Swedish PAID. The four-model of the Spanish PAID included item 15 (“feeling unsatisfied with your diabetes physician”) in a social support problems subscale, while the four-factor model of the Duch PAID included this item in a treatment problems subscale. The Greek and Malaysian versions of the PAID had a three-factor structure, both of which had different components of subscales compared to each other and to the Swedish version of the PAID. The Brazilian and German versions of the PAID fitted the four-factor structure, while the Kuwaiti AR-PAID fitted the five-factor structure (19, 20, 22, 26, 28–30, 33).

Our CFA was inadequately fit with one-factor and two-factor models. A one-factor structure had been found in the original version of the PAID, which was demonstrated by Welch et al. in addition to the Brazilian, Taiwanese, and Polish versions of the PAID (13, 20, 23, 32). A two-factor structure was reported in the Icelandic and Turkish versions of the PAID (21, 24). Miller and Elasy also identified a two-factor structure of the PAID in southern rural African-American women with T2DM (50). The Korean PAID was marginally fit with one-factor to four-factor models (27).

These discrepancies of the structures of these versions of the PAID could be related to differences in the cultural and clinical characteristics of the participants, the existing health care system and diabetic care services that might affect patients' perceptions about diabetes-distress, the use of exploratory factor analysis in some previous studies, and the use of different methods.

Convergent validity of the total AR-PAID scale was supported by the moderate-to-strong positive correlations with the PHQ-9 and GAD-7 scores, by the strong negative correlation with the WHO-5 score, and by the weak positive correlation with HbA1c levels. The AR-PAID displayed strong correlation with the PHQ-9, whereas both previous studies found moderate correlation with the PHQ-9 (32, 51, 52). The AR-PAID displayed moderate correlation with the GAD-7, which was similar to what was found in an Australian study (52). The AR-PAID correlated moderately and negatively with the WHO-5, and those results were consistent with those found in validation studies of the Turkish and Polish versions of the PAID (24, 32).

The total score of the AR-PAID has a weak positive correlation with HbA1c levels, similarly observed in previous studies (13, 19, 22, 23, 25–28, 30, 32). However, other studies found insignificant associations between versions of the PAID and HbA1c levels (20, 24, 29). This association was not assessed in the Kinyarwanda PAID, as the HbA1c test was not assessed systematically in the Rwandan patients with diabetes (31).

In our study, the emotional problem subscale had the highest correlation with depression, anxiety, wellbeing, and glycemic control (HbA1c levels) compared with other subscales. These findings might be related to the importance and relevance of the emotional problems subscale for evaluating the pivotal content of diabetes-stress (28, 37). Therefore, further study is needed to assess the psychometric properties of the short version of the AR-PAID.

The total AR-PAID scale had an excellent internal consistency (α = 0.96), which was in line with the reported findings from the original, Duch, Brazilian, Icelandic, Swedish, Turkish, Norwegian, Greek, Korean, Spanish, German, Malaysian, and Polish versions of the PAID (α ranged 0.90–0.96) (12, 13, 19–22, 24–30, 32). The Kinyarwanda version of the PAID demonstrated a good internal consistency (α = 0.88) (31), while the internal consistency analysis was not reported in the Taiwanese version of the PAID (23).

We found that the emotional problems subscale had demonstrated the highest Cronbach's alpha. This unsurprising finding might be the result of its homogenous construct, as well as inclusion of more items in this subscale: twelve items, compared to other subscales. The treatment problems subscale also had an excellent internal consistency (α = 0.94), which is inconsistent with the findings of the Korean PAID (α=0.54). Lee et al. had explained their finding as a result of the relative heterogeneity of its three items, which may be related to the presence of item 15 (27).

The total AR-PAID scale was found to have good 1-month test-retest reliability (ICC = 0.97), indicating the stability of the scale. Previous studies reported a satisfactory stability of the total PAID scale (19, 23, 25, 27), whereas the test–retest of it was not assessed in other studies (12, 13, 20–22, 24, 28–34, 50). We found that the AR-PAID subscales demonstrated satisfactory stability (ICC ranged 0.92–0.97). Snoek et al. (19) and Lee et al. (27) had both reported instability of their treatment problems subscale.

Our results provided sound support for the discriminant validity of the AR-PAID in terms of determining differences in diabetes-distress with patients' sociodemographic and clinical characteristics. We found older people with T2DM showed significantly higher diabetes-distress scores than other age groups, which might have been explained by how older people reported being usually concerned about the presence and seriousness of diabetes-related complications, dealing with complications, and controlling diabetes, as well as being anxious and having guilty sensations if they are not able to achieve good glycemic targets. However, other studies found that younger patients had higher scores on the PAID (19, 20, 24, 32). Female patients had significantly higher AR-PAID total scores than male patients. Similar findings have been found in previous studies (19, 20, 24–26, 32, 33).

In the current study, patients with longer disease duration and those who are on insulin-containing regimen reported significantly higher AR-PAID scores, which was similar to the findings of a Greek study (26). Patients with an increasing number of the diabetes-related complications showed significantly higher AR-PAID total scores, and this finding was in line with the findings from a validation study of the Turkish PAID (24). We did not find an association between the AR-PAID total scores and achieving good glycemic targets, though this might be due to the relatively few participants who were able to achieve good glycemic targets. Known-group validity was supported by the significant associations of the AR-PAID total scores with depression, anxiety, and general wellbeing.

To the best of our knowledge, this is the first study that assessed the validity and reliability of the AR-PAID among PHC patients in the Middle East and North Africa region. Our study included only PHC patients with T2DM, which limited our ability to generalize the results to patients with type 1 diabetes. Our study included a relatively small number of patients who were able to achieve good glycemic control, which restricted the group validity on this clinical variable. Lastly, the cross-sectional design of our study could not assess the responsiveness of this instrument.

## Conclusions

The AR-PAID scale is a valid and reliable instrument for assessing diabetes-distress among Egyptian PHC patients with T2DM. Further studies are needed to assess the responsiveness of the AR-PAID, to assess cultural adaptations of the AR-PAID in other Arabic-speaking patients with diabetes, and to assess the validity and reliability of short forms of the AR-PAID.

## Data Availability Statement

The raw data supporting the conclusions of this article will be made available by the authors, upon reasonable request.

## Ethics Statement

The studies involving human participants were reviewed and approved by Ethics Committee of Faculty of Medicine, Suez Canal University, Ismailia, Egypt (Ref No. 4277/2020). The patients/participants provided their written informed consent to participate in this study.

## Author Contributions

HS commenced the idea of this study, participated in the designing the study, wrote the manuscript draft, and approved the final version of the manuscript. SM participated in the designing of this study, collected the data, revised the manuscript, and approved the final version of the manuscript. SE participated in the designing of this study, analyzed the data, revised the manuscript, and approved the final version of the manuscript. MM and JS participated in the designing of this study, revised the manuscript, and approved the final version of the manuscript. AF designed this study, analyzed the data, revised the manuscript, and approved the final version of the manuscript. All authors contributed to the article and approved the submitted version.

## Conflict of Interest

The authors declare that the research was conducted in the absence of any commercial or financial relationships that could be construed as a potential conflict of interest.

## Publisher's Note

All claims expressed in this article are solely those of the authors and do not necessarily represent those of their affiliated organizations, or those of the publisher, the editors and the reviewers. Any product that may be evaluated in this article, or claim that may be made by its manufacturer, is not guaranteed or endorsed by the publisher.

## Abbreviations

AR-PAID: Arabic version of the Problem Areas in Diabetes
BMI: Body Mass Index
CFA: Confirmatory factor analysis
CFI: Comparative fit index
CI: Confidence interval
df: Degrees of freedom
DM: Diabetes mellitus
GAD-7: Generalized Anxiety Disorder Scale 7
GFI: Goodness-of-fit index
HbA1c: Glycated hemoglobin
HTN: Hypertension
ICC: Intraclass correlation coefficient
IQR: Interquartile range
PAID: Problem Areas in Diabetes
PAID-1: 1-item Problem Areas in Diabetes
PAID-5: 5-item Problem Areas in Diabetes
PHC: Primary Healthcare
PHQ-9: Patient Health Questionnaire 9
Rho: Spearman's Rank-Order Correlation
RMSEA: Root mean squared error of approximation
SD: Standard deviation
SPSS: Statistical Package for the Social Sciences
SRMR: Standardized root mean square residual
T2DM: Type 2 diabetes mellitus
TLI: Tucker Lewis Index
WHO: World Health Organization
WHO-5: 5-item World Health Organization Well-Being Index
χ^2^: Chi-square
CMIN/DF: Ratio of Chi-square value to the degrees of freedom.
