# Corrigendum: Psychometric properties of the arabic version of the problem areas in diabetes scale in primary care

**DOI:** 10.3389/fpubh.2022.994563

**Published:** 2022-07-29

**Authors:** Hazem A. Sayed Ahmed, Samar Farag Mohamed, Sally Fawzy Elotla, Mona Mostafa, Jaffer Shah, Ahmed Mahmoud Fouad

**Affiliations:** ^1^Department of Family Medicine, Faculty of Medicine, Suez Canal University, Ismailia, Egypt; ^2^Department of Public Health, Occupational and Environmental Medicine, Faculty of Medicine, Suez Canal University, Ismailia, Egypt; ^3^Department of Internal Medicine, Faculty of Medicine, Suez Canal University, Ismailia, Egypt; ^4^Medical Research Center, Kateb University, Kabul, Afghanistan

**Keywords:** diabetes-distress, PAID, primary healthcare, type 2 diabetes, Arabic

In the published article, there was an error in the article title. Instead of “**Psychrometric Properties of the Arabic Version of the Problem Areas in Diabetes Scale in Primary Care**,” it should be “***Psychometric* Properties of the Arabic Version of the Problem Areas in Diabetes Scale in Primary Care**.”

The authors apologize for this error and state that this does not change the scientific conclusions of the article in any way. The original article has been updated.

## Publisher's note

All claims expressed in this article are solely those of the authors and do not necessarily represent those of their affiliated organizations, or those of the publisher, the editors and the reviewers. Any product that may be evaluated in this article, or claim that may be made by its manufacturer, is not guaranteed or endorsed by the publisher.

